# Combination of high-fat/high-fructose diet and low-dose streptozotocin to model long-term type-2 diabetes complications

**DOI:** 10.1038/s41598-017-18896-5

**Published:** 2018-01-11

**Authors:** David André Barrière, Christophe Noll, Geneviève Roussy, Farah Lizotte, Anissa Kessai, Karyn Kirby, Karine Belleville, Nicolas Beaudet, Jean-Michel Longpré, André C. Carpentier, Pedro Geraldes, Philippe Sarret

**Affiliations:** 1 Département de Pharmacologie et Physiologie/Institut de Pharmacologie de Sherbrooke, Université de Sherbrooke, Québec, Canada; 2 Département de Médecine, Service d’Endocrinologie, Faculté de Médecine et des Sciences de la Santé, Université de Sherbrooke, Québec, Canada

## Abstract

The epidemic of type 2 diabetes mellitus (T2DM) is fueled by added fructose consumption. Here, we thus combined high-fat/high-fructose diet, with multiple low-dose injections of streptozotocin (HF/HF/Stz) to emulate the long-term complications of T2DM. HF/HF/Stz rats, monitored over 56 weeks, exhibited metabolic dysfunctions associated with the different stages of the T2DM disease progression in humans: an early prediabetic phase characterized by an hyperinsulinemic period with modest dysglycemia, followed by a late stage of T2DM with frank hyperglycemia, normalization of insulinemia, marked dyslipidemia, hepatic fibrosis and pancreatic β-cell failure. Histopathological analyses combined to [^18^F]-FDG PET imaging further demonstrated the presence of several end-organ long-term complications, including reduction in myocardial glucose utilization, renal dysfunction as well as microvascular neuropathy and retinopathy. We also provide for the first time a comprehensive µ-PET whole brain imaging of the changes in glucose metabolic activity within discrete cerebral regions in HF/HF/Stz diabetic rats. Altogether, we developed and characterized a unique non-genetic preclinical model of T2DM adapted to the current diet and lifestyle that recapitulates the major metabolic features of the disease progression, from insulin resistance to pancreatic β-cell dysfunction, and closely mimicking the target-organ damage occurring in type 2 diabetic patients at advanced stages.

## Introduction

Type 2 diabetes is a long-term metabolic disorder that represents a global public health challenge, affecting not only industrialized countries, but also increasing drastically in developing nations^1^. Importantly, patients with type 2 diabetes *Mellitus* (T2DM) display higher risk to develop severe complications, such as cardiomyopathy, nephropathy, neuropathy and retinopathy^2–5^. Over the years, it has become more and more evident that the development of T2DM is fueled by bad diets and unhealthy lifestyles^6^. In particular, intake of added sugar, and especially fructose consumption as from sucrose or high-fructose corn syrup found in soft drinks and sugar-sweetened foods, has been linked to diabetes-related metabolic complications^7–9^. Indeed, fructose which is a highly lipogenic monosaccharide promotes insulin resistance, impaired glucose metabolism, dyslipidemia, hepatic fibrosis and steatosis, as well as both cardiac and renal dysfunctions^10–12^.

Animal models can deliver invaluable information to our knowledge of the pathophysiology of T2DM complications^13,14^. However, the translational value of these animal models can be further enhanced by the reduction of the gap between preclinical and clinical research. This notably requires a better understanding of the role of environmental factors that influence the spread and severity of the disease, including diet and lifestyle changes. Although genetically modified animal models reproduce many metabolic and organic failures occurring in T2DM patients, the development of experimentally-induced diabetic animals has further helped to identify the pathobiology and metabolic features associated with the different stages of the T2DM disease progression in humans^13,15,16^. For instance, a high fructose diet is often conjugated with a high fat diet (HF/HF) to induce T2DM in rodents^17–19^. However, this model refers only to the early stage of T2DM pathology or prediabetes since animals never develop β-cell failure as observed in advanced stages of T2DM in humans. In 2000, Reed *et al*. proposed to combine high fat diet (HFD) with a single injection of streptozotocin (Stz) to initiate the β-cell dysfunction in rodents^20^. Now, a growing number of animal studies demonstrates that a low dose of Stz combined with HFD^21–24^, sucrose^25^, or fructose^26^ can recapitulate certain features of diet-induced T2DM seen in humans.

Here, we propose to combine the main diet stressors encountered in the human population, namely high fat/high fructose diet, with multiple low-dose injections of Stz to study the long-term complications associated with the development of T2DM. To determine if this new animal model is advantageous to closely mimic the human condition, we therefore monitored during 56 weeks the effects of this HF/HF/Stz regimen on the time-course metabolic changes as well as on the end-organ damage.

## Methods

### Animals and treatment

**Figure 1:**
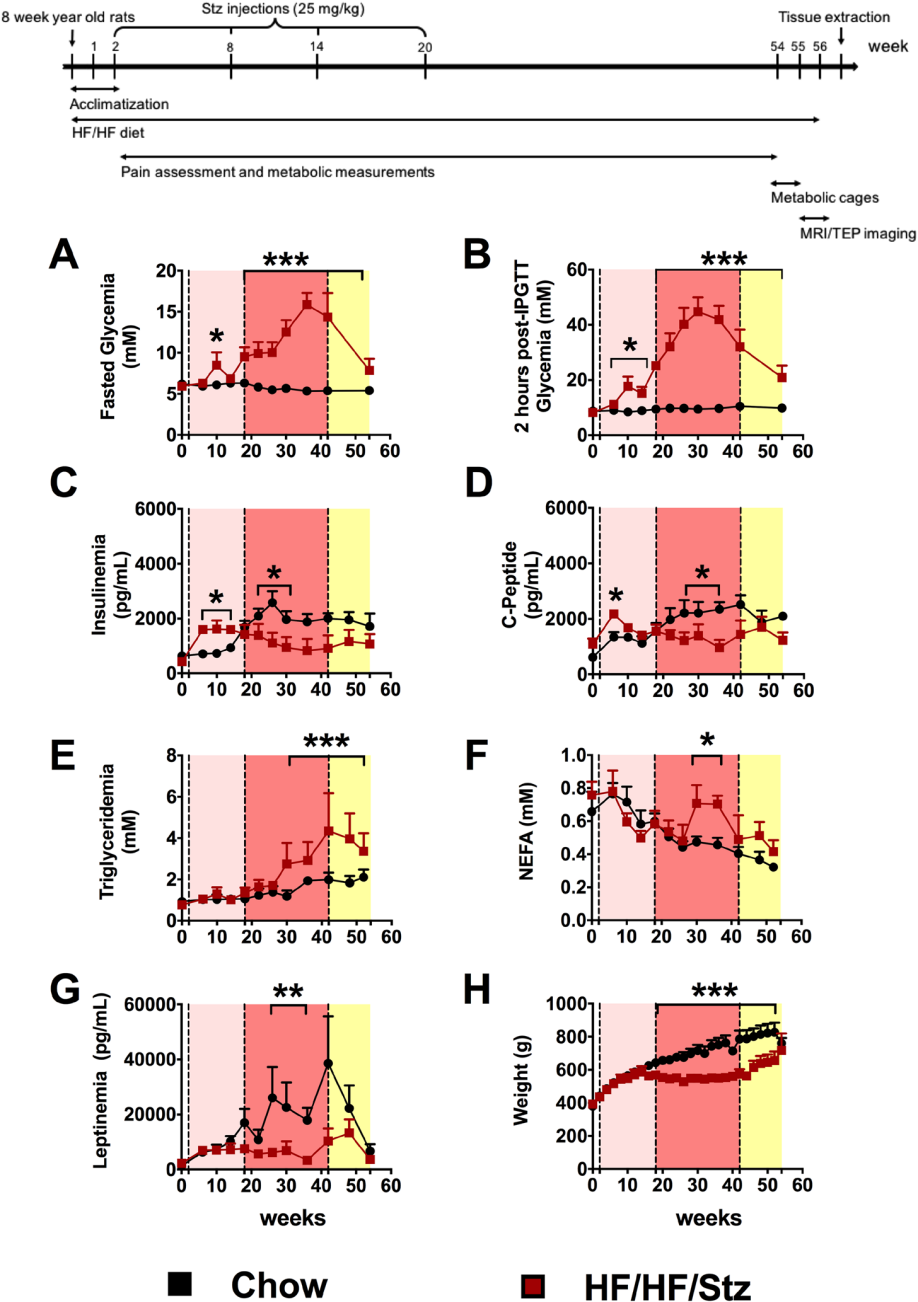
Effect of HF/HF/Stz regimen on glucose and lipid metabolism. The time-course metabolic changes in HF/HF/Stz rats reveal the presence of three distinct phases. An early phase between 2 and 18 weeks (pink) characterized by both fasting (**A**) and 2 h post-IPGTT hyperglycaemia (**B**), insulin resistance (**C**) and C-peptidemia (**D**). The time-course of early metabolic changes does not reveal any significant changes in triglyceridemia (**E**), non-esterified fatty acid (NEFA) levels (**F**) and leptinemia (**G**) and weight gain (**H**) in the HF/HF/Stz group, when compared to normal diet-fed control rats. The second phase (week 18 to 42; dark pink) reveals a frank hyperglycaemia associated with a decline in insulin production. The significant increase in plasma levels of triglycerides and NEFA is accompanied by a decrease in body weight and leptinemia. During the late phase (week 43 to 56; yellow), both fasting and post-IPGTT glucose levels decrease but remain significantly higher than in the Chow-fed group. Finally, weight, leptinemia, and NEFA levels are comparable in both groups. Data are compared using a two-way ANOVA followed by Holm-Sidak multiple comparisons test and expressed as mean ± SEM; *P < 0.05; **P < 0.01 and ***P < 0.001, compared with control fed rats.

Eight-week-old male Wistar rats (Charles River, Québec, CA) were housed two per cage with a 12:12-h light-dark cycle at constant temperature (22 °C). Control group (Chow; n = 6) was fed *ad libitum* during 56 weeks with regular chow (Rodent Laboratory Chow 5001, Purina, USA), whereas the high-fat/high-fructose group (HF/HF; n = 6) was fed with HF/HF diet (TD 05482 rat chow, Teklad, USA), containing 46.5 wt% fructose and 25.7 wt% lard for 56 weeks. Both groups had free access to water. Two weeks after arrival, juvenile HF/HF rats were injected intraperitoneally with a small dose of streptozotocin (Stz; 25 mg/kg, Sigma, CA), according to^11^. As shown in Fig. 1, STZ injections were repeated three times every six weeks after the initial injection in order to chronically stress Langerhans islets and induce a frank hyperglycaemia. The cumulative dose does not excess 100 mg/kg over the 56-week experimental period. As a control group, rats received vehicle injections (sodium citrate buffer, 2%). Throughout the experiment, animals were weighted each week and post-prandial glycaemia was measured (Glucometer 4 Accu-chek, CA). The experiments were conducted in accordance to *The University of Sherbrooke’s* local research ethics committee, guidelines of the Committee for Research and Ethical Issues of IASP and ARRIVE guidelines.

### Intraperitoneal glucose tolerance test

Over 54 weeks, we performed the intraperitoneal glucose tolerance test (IPGTT), as previously described^27^. Fasted glycaemia and post-IPGTT glycaemia were measured (Accu-chek, CA). Plasma insulin, leptin and C-peptide were measured by ELISA (Millipore, USA). Plasma triglycerides and non-esterified Fatty Acid (NEFA) were measured, as previously described^28^.

### Energy consumption

At week 54, animals were housed for seven consecutive days in metabolic cages with full access to food and water. Each day, animals were weighted, volume of water intake and amount of food consumed were calculated and the 24 h-urine and -feces collections were measured.

### Pain assessment

Three behavioral tests were used to assess mechanical allodynia as well as mechanical and thermal hyperalgesia over the 54-week period. Mechanical allodynia was performed according to the method described by Chaplan *et al*. allowing to assess the 50% paw withdrawal threshold following application of tactile non-noxious stimuli. Mechanical hyperalgesia (Randall-Selitto test) was determined using an analgesia meter (Ugo Basile, Stoelting, USA) that applies a linearly increasing pressure (16 g/s) on the medial dorsum of the hind paw. Mean of four consecutive measures (cut-off 200 g) were assessed on each paw to determine the mechanical withdrawal threshold (g). Thermal hyperalgesia (Hargreaves’ Method) was evaluated using the Plantar test apparatus (Ugo Basile, Varese, It). After a 1 h habituation period, the plantar surface of the hind paw was exposed three times (cut-off time 20 s) to a controlled infrared noxious radiant heat stimulus allowing the measurement of the precise withdrawal latency (s).

### *In vivo* Imaging

#### MRI and TEP acquisition procedures

MRI was performed using a small-animal 7 Tesla scanner (Varian, Palo Alto, USA) equipped with 205/120 Magnex gradient coils, a transmit volume coil and rat head surface coil (Rapid Biomedical, Columbus, USA). Rats were anaesthetized with 3% (induction) and 1.5% (maintenance) isoflurane in oxygen. A feedback-controlled animal warm-air heater system was used to maintain body temperature and the respiration rate was continuously monitored (SA Instruments Inc, USA). Proton density-weighted images were acquired using a fast spin-echo pulse sequence to generate brain volume. T2 brain maps obtained from proton density-weighted images were acquired using a fast spin-echo pulse sequence (with a repetition time of 3.600 ms, an effective echo time of 12 ms, a number of echoes of 8 and a number of averaging of 8. We acquired 35 axial slices 700 µm thick with a field of view of 32 × 32 mm in a matrix of 256 × 256 resulting in an in-plane resolution of 125 × 125 µm.

After MRI scanning, rats were transferred in the Triumph™ PET/CT dual modality imaging platform (Gamma Medica, Inc., Northridge, CA, USA), consisting of a LabPET™ avalanche photodiode-based digital PET scanner with a 7.5 cm axial field-of-view capable of achieving an isotropic spatial resolution of 1.2 mm and a detection efficiency of 2.1% with an energy window setting of 250–650 keV. Rats were also equipped for ECG recording, centered in the scanner for heart imaging before receiving a first caudal injection of 20 MBq [^11^C]-acetate. 20 min-dynamic PET scans were then acquired before moving the rat in the scanner for brain imaging. Hence, a caudal injection of approximately 30 MBq of [^18^F]-FDG was applied followed by a 30 min-dynamic scan. At equilibrium, a three bed static acquisition of 5 min each was recorded for [^18^F]-FDG uptake within brain, heart and kidney. Finally, CT images were acquired from the high-resolution X-ray computed tomography (CT) modality.

Images were reconstructed using the Triumph™ PET/CT software. ECG-gated dynamic [^11^C]-acetate series of 27 frames (12 for 10 s, 8 for 30 s, 6 for 90 s and 1 for 300 s) were reconstructed using 3-D MLEM algorithm using the following parameters: 20 iterations, span of 63, field of view of 80 mm with a final matrix resolution of 160 × 160 × 128 and a voxel size of 0.5 × 0.5 × 0.597 mm. Brain dynamic [^18^F]-FDG images were reconstructed using the same protocol but we generated 32 frames (10 for 5 s, 7 for 10 s, 6 for 30 sec, 6 for 120 s, 2 for 240 s and 1 for 300 s). Static [^18^F]-FDG images were reconstructed with the same parameters but we generated one frame. CT images were acquired from the high-resolution X-ray computed tomography (CT) modality. Scans were performed at 60 kVp and 230 µA using 512 projections in fly mode with a 84.57 mm in field of view. Images were obtained using the standard FBP kernel analytical reconstruction algorithms, providing an isotropic image of 512 slices with a final resolution of 0.165 µm isotropic.

#### Cardiac imaging analysis

For dynamic cardiac imaging, a 20-min dynamic acquisition with [^11^C]-acetate was done to determine both systolic and diastolic volume, as previously described^11^. For analysis of ventricular function, PET data from [^11^C]-acetate images were obtained as a series of eight ECG-gated frames. Corridor4DM version 5.2 software (Segami, Invia) was used for reorientation and to compute left ventricular volumes. For myocardial oxidative index, analysis was done by drawing regions of interest (ROIs) on short-axis images on Amide software v.1.0.5 and input curves were extracted by means of a ROI drawn on the left ventricular cavity blood pool in summed last-frame images to seek better contrast. Myocardial oxidative index was calculated by using a three-compartment kinetic model that estimates the generation of CO_2_ from the citric acid cycle in the myocardium using the *k*2 value^11^.

#### Brain imaging analysis

Each MRI scan was normalized to rat T2 brain template available from PMOD (http:/doc.pmod.com/pbas/4997.htm) using Statistical Parametric Mapping software (SPM, Wellcome Department, UK) to project them on the same space. Normalized MRI T2 images were then co-registered with its respective CT scan and matrix transformations were applied on dynamic as well as static PET brain images. Prior to statistical analysis, a brain mask was applied over the PET scan to exclude extracerebral regions. Each of the [^18^F]-FDG scans was finally individually smoothed using Kernel Gaussian filter 0.6 mm FWHM.

Differences in [^18^F]-FDG uptake between Chow and HF/HF-Stz groups were assessed using a voxel-by-voxel two-sample paired t-test. Global uptake differences between brain scans were adjusted using the “proportional scaling” SPM option. A significance level threshold of 0.05 (False Discovery Rate correction) and a minimum cluster size of 200 voxels were selected.

#### Calculation of Standard Uptake Values of [^18^F]-FDG in heart and kidney

Static [^18^F]-FDG images were analyzed with an OsiriX viewer (version 4.0, 64 bit; OsiriX). To determine the level of variation in radiotracer uptake, the mean standardized uptake values (SUVmean) were calculated according to the following formula, as previously described:

$$SUV\,mean=mean\,uptake\,value/(dose\,injected\,[MBq]\times animal\,weight\,[kg])$$

Regions of interest (ROI) have been drawn on three consecutive slices for the heart and kidneys, then raw data were extracted from these ROIs and the SUV means were calculated.

### Histology and Immunofluorescence

At the end of the experiment, rats were anesthetized with isoflurane (3–5%) and transcardially perfused with 500 mL of 4% paraformaldehyde (PFA) in 0.2 M PBS (pH 7.4). Liver, kidney, heart, sciatic nerve, dorsal root ganglion (DRG), spinal cord and eyes were then collected, post-fixed for 24 hours and finally embedded in paraffin for histological analysis. Paraffin-embedded sections of pancreas, liver, kidney and eyes (5 µm) were generated and stained using hematoxylin-eosin-safran (HES).

#### Pancreas

Image acquisitions have been performed on an automated platform Nikon Microscope C2+ and NIS-Elements Advanced Research software. Quantity and surface of Langerhans’ islets were automatically assessed using the difference within the contrast between Langerhans’ islets and acini in HES stained sections using the same threshold.

#### Kidney

Glomerular hypertrophy was assessed on HES specimen by measuring glomerular surface for each animal using Image J. Mesangial cell expansion was assessed using the staining of collagen IV on consecutive kidney sections. Sections were incubated overnight at 4 °C with a primary anti-collagen IV (1/500, Rabbit Novus biological, CA) and then processed using Dako streptavidin-biotin-peroxidase kit according to the manufacturer’s instructions (PK-6100, Vector Laboratories, USA). Quantification of collagen IV was realized by counting the number of positive pixels in the mesangium divided by the total area of each glomerulus by adjusting the threshold, permitting a binary analysis using ImageJ (NIH, Bethesda, MD).

#### Liver and heart fibrosis

Liver and heart sections were stained with Sirius red (0.5% in saturated picric acid) to detect collagen fibers^29^. On each vessel in the liver, the number and intensity of pixels of interstitial collagen were determined with Adobe Photoshop CS3 software and divided by the perimeter of the vessel, as previously described^30^.

#### Retinal tissue

Cross-sectional histological analysis of retinal tissue was also performed. Furthermore, eye sections were blocked with 10% goat serum for 1 h and were exposed to primary antibody CD31 (BD Bioscience 558736) (1:50) overnight, followed by incubation with secondary antibody Alexa-488 conjugated anti-rat IgG to reveal the presence of capillaries. Sections were mounted with Vectashield with 4,6-Diamidino-2-phenylindole (DAPI) (Vectors Laboratories). Vessel count was performed and normalized by the periphery of the retina (mm). Images were captured on a Nikon eclipse Ti microscope. All images were taken at the same time under identical settings and handled in Adobe Photoshop similarly across all images.

#### Dorsal root ganglia and spinal cord

Cryostat sections of dorsal root ganglia (DRG) were incubated overnight at 4 °C with either the anti-Substance P or anti-calcitonin gene-related peptide (CGRP) antibodies (1/500, Guinea Pig anti-SP and anti-CGRP, Neuromics, USA). Then, sections were incubated with FITC-conjugated secondary antibodies (1:1000, Invitrogen Molecular Probes, USA) for 2 h at room temperature. Non-specific staining was determined by excluding the primary antibody whereas the auto-fluorescence was chemically bleached using cupric sulfate (10 mM) in ammonium acetate buffer (50 mM, pH 5.0). Spinal cord sections were incubated overnight at 4 °C with either the anti-GFAP (1/1.000, Chicken anti-GFAP, Millipore, CA) or anti-IBA1 (1/500, rabbit anti-IBA1, Wako, USA) antibody using the same protocol. Additional dorsal horn spinal cord sections were generated to evaluate c-Fos expression. Sections were incubated with a primary c-Fos antibody (1/1000, Rabbit-anti c-Fos, Abcam, CA) and processed using the Dako streptavidin-biotin-peroxidase kit. To provide valid quantification results, all images of each spinal cord were captured under identical exposure times using a Leica DM4000 microscope equipped with a Leica DFC350FX camera. Positive cells were manually counted by a blinded experimenter from a set of pictures containing both experimental groups (Chow and HF/HF/Stz), including the previously randomized negative control. For quantitative analysis, three images per DRG or spinal dorsal horn per rat were acquired with a minimum of five animals per staining group. DRG tissue sections were counterstained with DAPI to reveal cell nuclei. The immunoreactivity was expressed as percentage of all cells counted.

#### Electron microscopy of sciatic nerves

Fixed sciatic nerves were washed in cacodylate buffer, post-fixed for 2 h at 4 °C in buffered osmium tetroxide, dehydrated, and embedded in Epon Araldite (Sigma Aldrich, CA). Ultra-thin sections (70 nm) were cut with an ultramicrotome (UC6, Leica, CA) and mounted onto copper grids. Sections were then stained with uranyl acetate and lead citrate, observed with a Hitachi H-7650 transmission electron microscope (Hitachi, Elexience, CA) and acquired with a CCD AMT HR camera (1024 × 1024 pixels; Hamamatsu, CA).

### Statistical analysis

All data were analyzed using GraphPad Prism 6.02 software. The data are presented as mean ± standard error of the mean (S.E.M.). Differences in pre- and post-IPGTT glycaemia, insulinemia, C-peptidemia, weight gain, triglyceridemia, leptinemia, non-esterified acids and pain thresholds were evaluated using a two-way ANOVA followed by Holm-Sidak multiple comparisons test. The number of c-Fos-immunoreactive cells was analyzed using a Kruskal-Wallis test (one-way ANOVA on rank) followed by the Dunn’s multiple comparisons test. All other data including histology, cardiac imaging, metabolic information, behavioral assessment (area under the curve) did not assume a Gaussian distribution (d’Agostino & Pearson omnibus normality test) and were consequently analyzed using a Mann-Whitney comparison test (U-test). The level of statistical significance was set at P < 0.05 (*).

## Results

### Effects of HF/HF/Stz regimen on the time-course metabolic changes

Assessment of fasting and post-IPGTT glycaemia, insulinemia, C-peptidemia, triglyceridemia, non-esterified acid (NEFA) and leptinemia in both HF/HF/Stz and Chow groups revealed the presence of contributing symptoms to the development of T2DM. In rats submitted to the HF/HF diet and multiple low-doses of streptozotocin, we first observed an initial phase between 2 and 18 weeks (pink section) where animals displayed moderate glucose intolerance characterized by both fasting and 2 h post-IPGTT hyperglycemia (Fig. 1A,B, P < 0.05). This impaired glucose metabolism was also associated to insulin resistance as observed by higher plasma levels of insulin and C-peptide (Fig. 1C,D, P < 0.05). During this first phase, weight gain as well as triglyceridemia, NEFA and leptin levels were not modified, when compared to normal diet-fed control rats (Fig. 1E–H).

**Figure 2:**
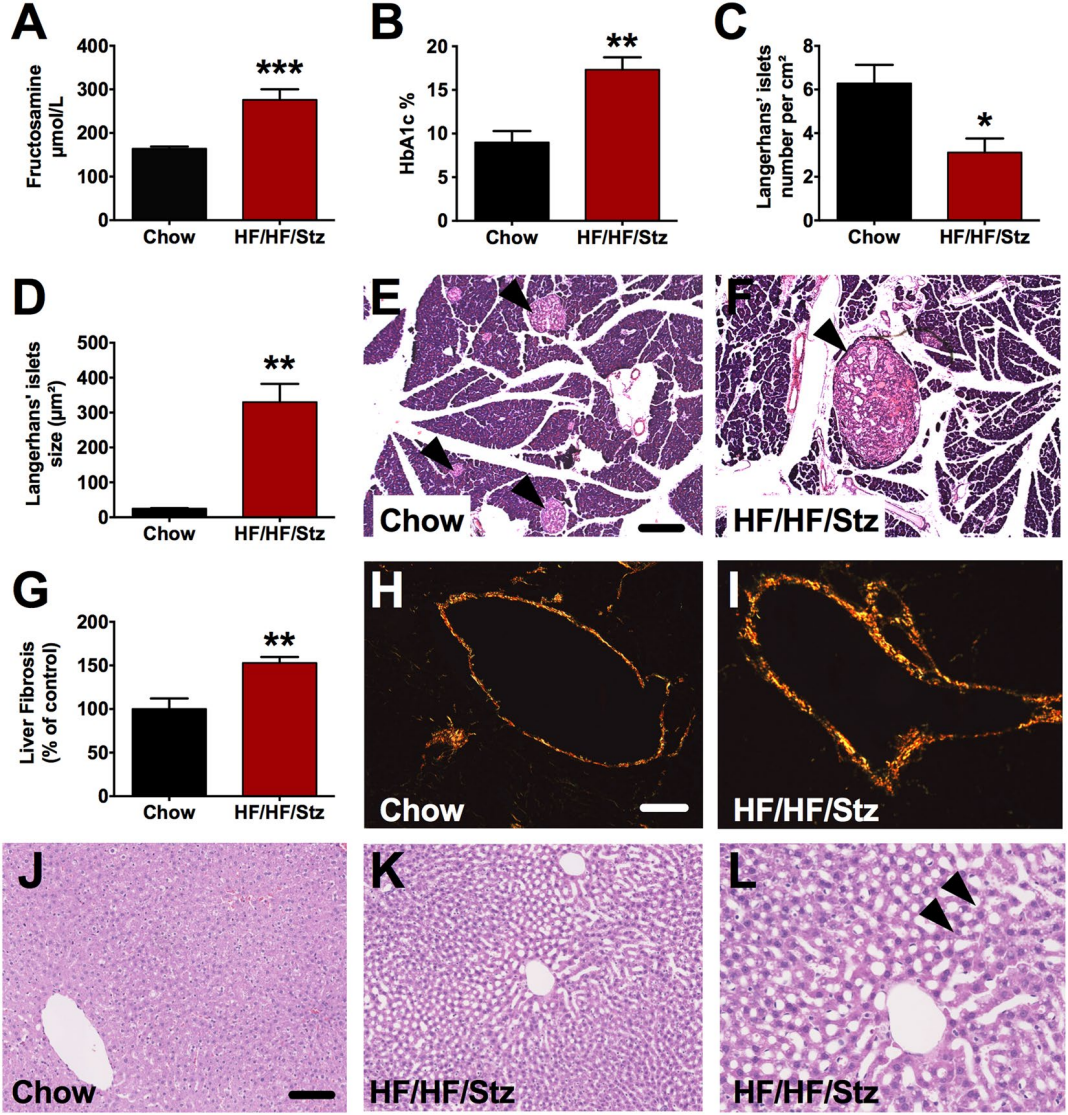
Effect of HF/HF/Stz regimen on pancreatic and liver function. During the second phase (week 33), higher levels of plasma fructosamine (**A**) and glycated hemoglobin (HbA1c; **B**) are measured. At the end of the experiment (week 56), the number of Langerhans’ islets significantly decreases in HF/HF/Stz rats (**C**) whereas their size increases drastically (**D**). Hematoxylin and eosin staining revealed that the Langerhans’ islets are hyperplasic, congestive and hemorrhagic in the HF/HF/Stz group (**F**, arrowheads) compared to controls (**E**, scale bar 100 µm). Sirius red F_3_BA staining indicate the presence of liver fibrosis (Type I and III collagen) in the HF/HF/Stz group (**H–I**, scale bar 50 µm). Liver steatosis was evaluated by H&E staining in both HF/HF/Stz and control animals (**J–K**). We noticed the presence of a large number of lipid vacuoles in hepatocytes of HF/HF/Stz rats (arrowheads, **L**, scale bar 65 µm). Data in (**A–D** and **G)** are compared using an exact two-tailed Mann-Whitney U-test because a Gaussian distribution is not assumed here (d’Agostino-Pearson omnibus normality test). Data are expressed as mean ± SEM; *P < 0.05; **P < 0.01 and ***P < 0.001, compared with normal diet-fed control rats.

Between 18 and 42 weeks (red section), we observed a transition from prediabetes and/or insulin resistance to frank type 2 diabetes. Indeed, this second phase was characterized by a frank fasting and post-IPGTT hyperglycemia (Fig. 1A,B, P < 0.001), high triglyceride concentrations (Fig. 1E, P < 0.001), higher levels of plasma fructosamine (Fig. 2A, P < 0.001) and glycated hemoglobin (HbA1c) (Fig. 2B, P < 0.01), as well as pancreatic beta-cell dysfunction leading to insulinopenia and decrease in C-peptide levels (Fig. 1C,D, P < 0.05). During this second phase, we also found a significant increase in plasma NEFA levels (Fig. 1F, P < 0.05) concomitant with a reduction in circulating leptin, thus reflecting disruption of the adipose tissue balance (Fig. 1G, P < 0.01). This adipose tissue lipolysis was further accompanied by a decrease in body weight gain (Fig. 1H, P < 0.001), which may rely on glucose loss in the urine (Data not shown). In parallel, normal chow-fed rats displayed hyperinsulinemia and hyperleptidemia compared to baseline without any change in glucose tolerance (Fig. 1).

After 42 weeks (yellow section), fasting and post-IPGTT glycaemia decreased but remained significantly higher than levels of the chow-fed group (Fig. 1A,B, P < 0.05). Likewise, elevation of plasma triglycerides further confirmed the presence of dyslipidemia (Fig. 2B, P < 0.001). Insulin levels remained below than the control fed-rats whereas leptin and NEFA levels (Fig. 2A–D) of all HF/HF/Stz-treated rats were comparable to those of chow-fed animals. These metabolic changes were further accompanied by target-organ damage and cardiovascular complications as described below.

### Effects of HF/HF/Stz regimen on endocrine pancreatic and liver functions

Metabolic adaptation to the HF/HF/Stz regimen was associated with defects in pancreatic β-cell function. Indeed, morphometric analyses of pancreatic sections revealed that the number of Langerhans’ islets decreased significantly in the HF/HF/Stz group in accordance with the β-cell toxic effect of Stz (Fig. 2C, P < 0.05). In the meantime, the size of Langerhans’ islets increased drastically, when compared to controls (Fig. 2D, P < 0.01). Histopathological examination of the pancreas also revealed that the Langerhans’ islets were hyperplasic, congestive and hemorrhagic, thus explaining how the endocrine pancreas may maintain its production of insulin in the HF/HF/Stz group (Fig. 2E,F).

Within the liver, image analysis of collagen using Sirius red staining highlighted a significant increase in fibrosis in the HF/HF/Stz group (Fig. 2G–I, P < 0.01). Furthermore, H&E staining revealed the presence of a large number of lipid vacuoles in hepatocytes (Fig. 2J–L). The accumulation of these vacuoles supports the existence of hepatic steatosis in HF/HF/STZ rats. Finally, we did not notice the presence of systemic inflammation since plasma IL-6 and TNF- α levels were similar between diabetic and control rats (Data not shown).

### Effects of HF/HF/Stz regimen on the development of cardiomyopathy

**Figure 3:**
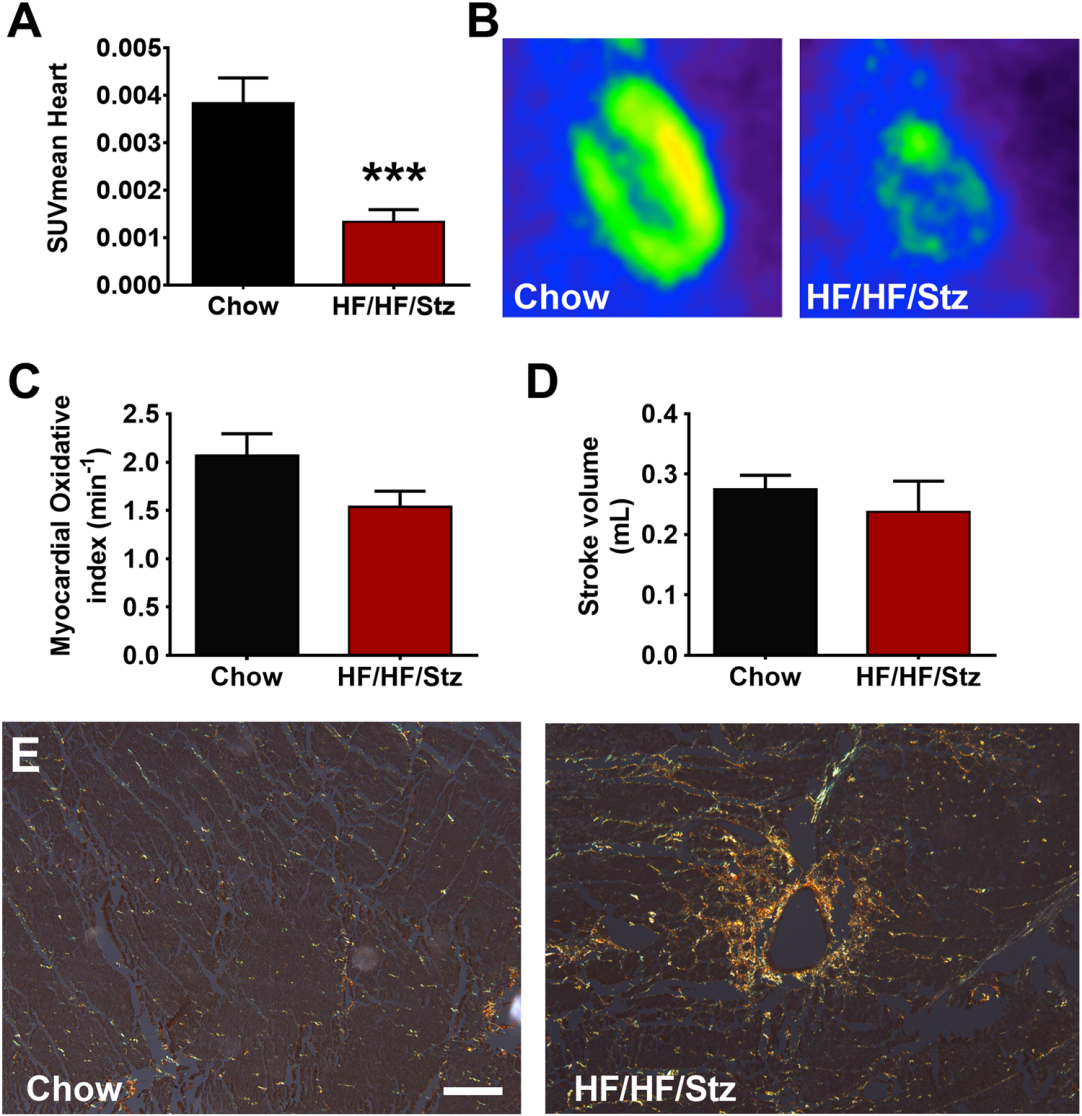
Effect of HF/HF/Stz regimen on cardiac function. Myocardial glucose uptake is drastically decreased in the HF/HF/Stz group, as observed 30 min after a bolus intravenous injection of ^18^Fluorodeoxyglucose (**A**,**B**). As determined by µPET [^11^C]-acetate cardiac imaging, a trend towards decreased in both myocardial oxidative index and stroke volume is noticed in rats receiving enriched diets after 56 weeks compared to controls (**C**,**D**). Data are expressed as mean ± SEM; ***P < 0.001, compared with rats receiving a standard chow diet; analyzed using a two-tailed Mann-Whitney U-test. An increase in heart fibrosis (Type I and III collagen) is observed by Sirius red F_3_BA staining in the HF/HF/Stz group (**E**, scale bar 50 µm).

Insulin resistance plays a critical role in diabetic cardiomyopathy. Here, we found using µ-PET that HF/HF/Stz rats displayed profound reduction in myocardial glucose utilization. Indeed, a significant decrease in myocardial [^18^F]-FDG uptake was observed in HF/HF/Stz *versus* control rats (Fig. 3A,B, P < 0.001). As assessed by µPET [^11^C]-acetate cardiac imaging, we further observed at week 56 a trend towards reduction in myocardial oxidative index and stroke volumes in HF/HF/Stz rats compared to controls (Fig. 3C,D). Finally, the presence of myocardial fibrosis was detected by Sirius Red staining in HF/HF/Stz rats, as observed by the increased collagen deposition (Fig. 3E).

### Effects of HF/HF/Stz regimen on renal function

**Figure 4:**
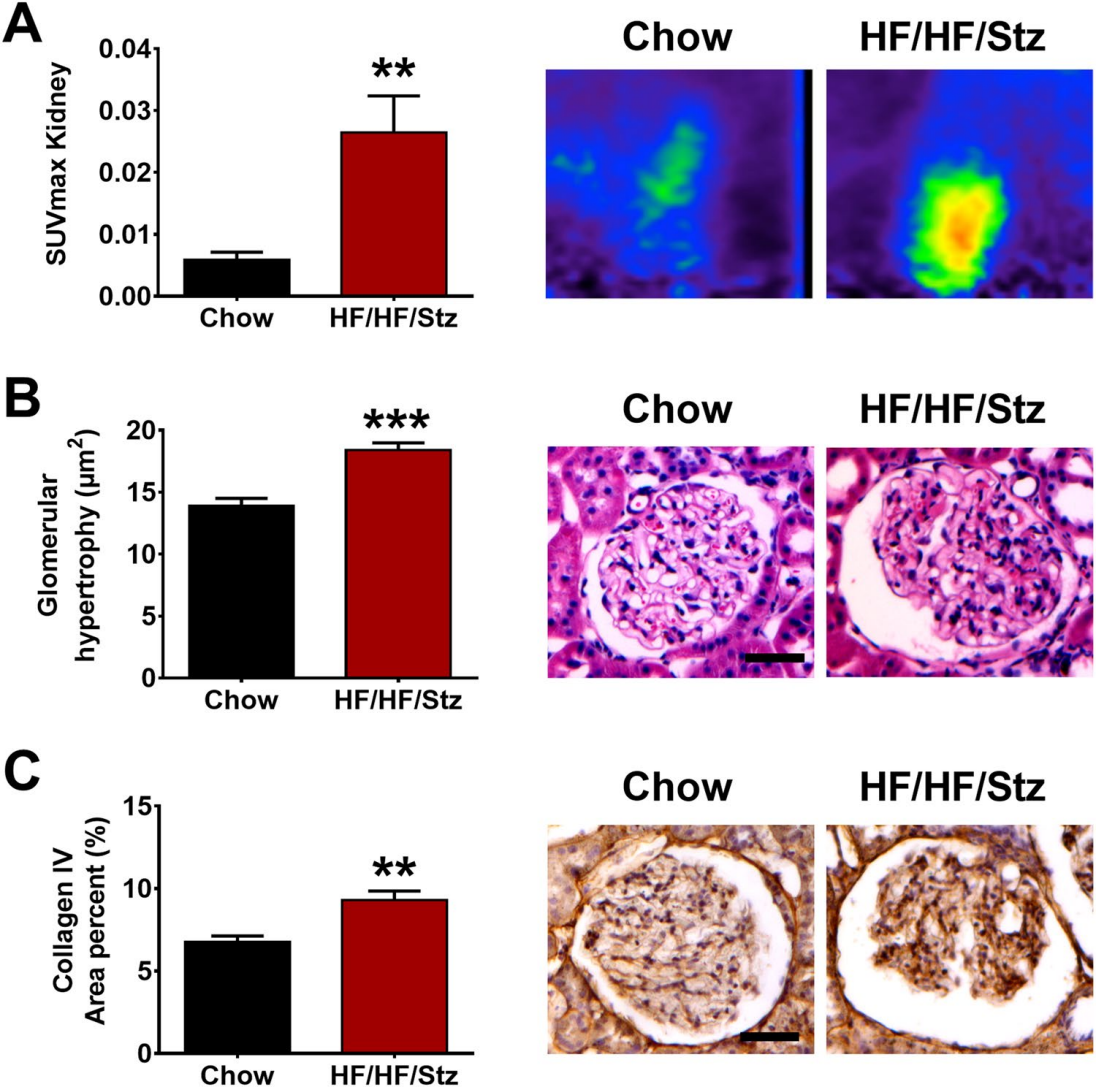
Effect of HF/HF/Stz regimen on kidney function. Renal dysfunction is first observed in the HF/HF/Stz group by measuring the [^18^F]-FDG uptake using µPET imaging (**A**). The presence of glomerular hypertrophy and kidney fibrosis, as determined respectively by H&E or collagen IV staining confirms the signs of kidney failure (**B**,**C** scale bar 50 µm). Data are expressed as mean ± SEM; **P < 0.01 and ***P < 0.001, compared with normal diet-fed control rats; analyzed using a two-tailed Mann-Whitney U-test.

At week 54 after initiation of the diet, detailed analysis of kidney structure and function revealed renal dysfunction. Indeed, a significant increase in [^18^F]-FDG uptake by kidneys, relating a higher filtration rate was observed in the HF/HF/Stz group (Fig. 4A, P < 0.01). Furthermore, kidney histological analysis showed significant glomerular hypertrophy (Fig. 4B, P < 0.001), larger Bowman’s capsule space (0.033 ± 0.004 µm^2^ *versus* 0.060 ± 0.005 µm^2^, not shown, P < 0.01) and mesangial cell expansion, as revealed by the accumulation of collagen IV in the HF/HF/Stz group (Fig. 4C, P < 0.01).

**Figure 5:**
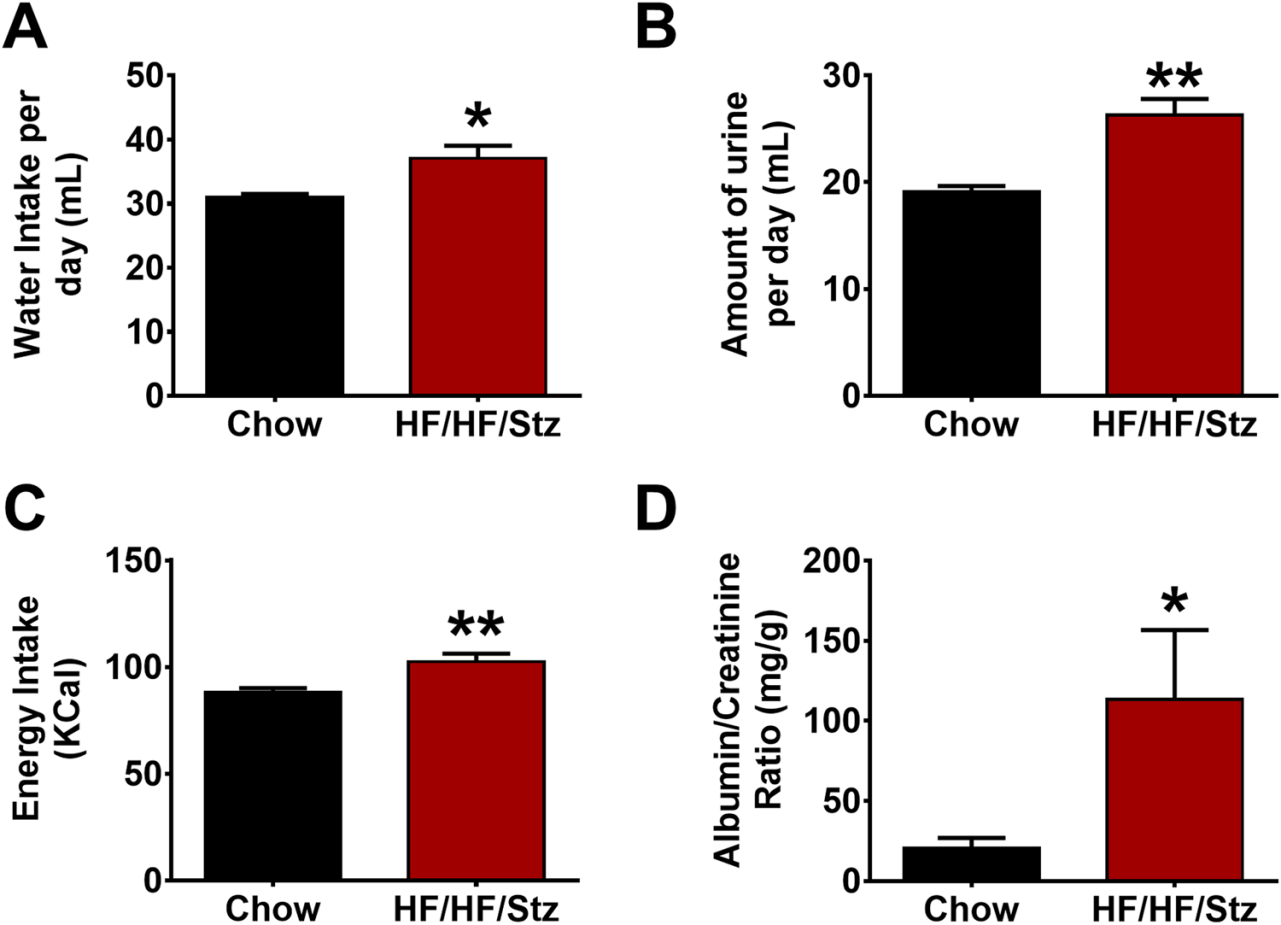
Effect of HF/HF/Stz regimen on renal function. At week 55, increases in water consumption (**A**) and urine volume (**B**) are observed in HF/HF/Stz rats. Furthermore, HF/HF/Stz-treated rats exhibit higher energy intake (**C**) than control fed-rats. The albumin/creatinine ratio (ACR) is also significantly increased in diabetic rats (**D**). Data are expressed as mean ± SEM; *P < 0.05; **P < 0.01, compared with normal diet-fed control rats; analyzed using a two-tailed Mann-Whitney U-test.

Rats were also housed individually for seven days in metabolic cages in order to measure daily feed and water consumption and determine biochemical parameters in urine samples. A significant increase in both water intake (Fig. 5A, P < 0.05) and 24 h urine volume (Fig. 5B, P < 0.01) was found in the HF/HF/Stz group. Total energy intake, determined from the energy content in each diet and mass consumed also indicated that HF/HF/Stz-treated rats exhibited higher energy intake compared to control fed-rats (Fig. 5C, P < 0.01). Importantly, we also found that the albumin/creatinine ratio (ACR) used as a marker of renal dysfunction was significantly elevated by 5-folds in diabetic rats as compared to control rats (Fig. 5D, P < 0.05). Urine analyses further demonstrated significant lower pH (6.9 ± 0.29 versus 5.3 ± 0.3, P < 0.01), presence of red blood cells, and signs of glycosuria in HF/HF/Stz rats (Data not shown).

### Effects of HF/HF/Stz regimen on diabetic retinopathy

**Figure 6:**
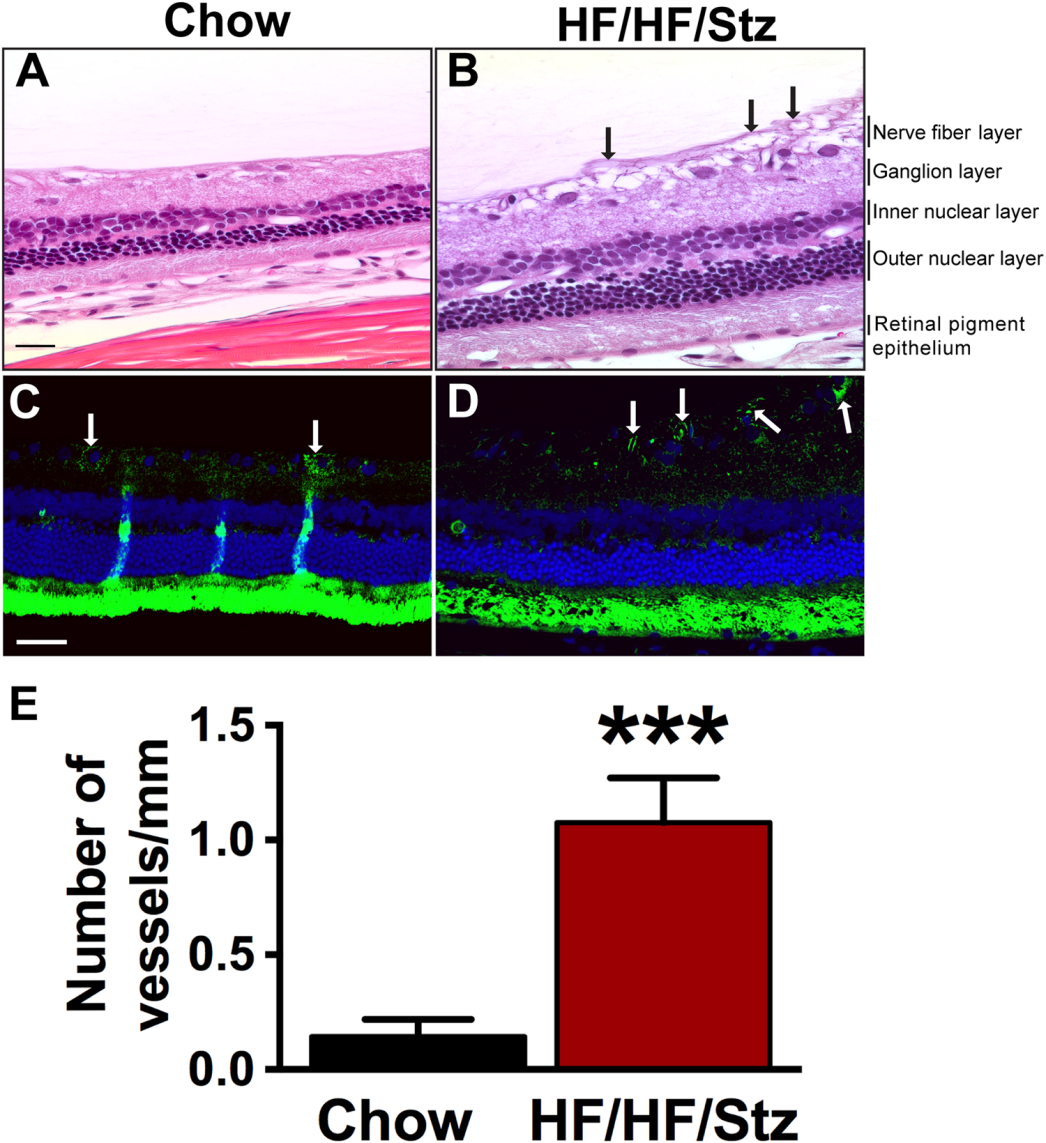
Effect of HF/HF/Stz regimen on the development of diabetic retinopathy. Cross-sectional histological analysis of retinal tissue reveals the presence of neovascular complications (**A**,**B** black arrows, scale bar 20 µm) as well as new blood vessel formation using endothelial cell marker (CD31) in HF/HF/Stz rats (**C**,**D**, white arrows, scale bar 50 µm). A higher number of vessels/mm in the nerve fiber layer is also observed in HF/HF/Stz rats (**E**, P < 0.001). Data are expressed as mean ± SEM; ***P < 0.001, compared with normal diet-fed control rats; analyzed using a two-tailed Mann-Whitney U-test.

Along with the development of nephropathy, we also observed other microvascular complications, such as diabetic retinopathy. Indeed, HF/HF/Stz rats exhibited signs of cataracts and blindness as early as week 20. Accordingly, cross-sections of the retina revealed the presence of morphological lesions, including thickening of the retinal parenchyma and pathological neovascularization with presence of endothelial cell marker (CD31) within the parenchyma, which are both characteristic of diabetic retinopathy (Black arrows in Fig. 6A and white arrow in Fig. 6B). Quantitative analysis also demonstrated the presence of a higher number of vessels per mm in the nerve fiber layer in HF/HF/Stz rats compared to control animals (Fig. 6E, P < 0.001).

### Effects of HF/HF/Stz regimen on the development of peripheral neuropathy

**Figure 7:**
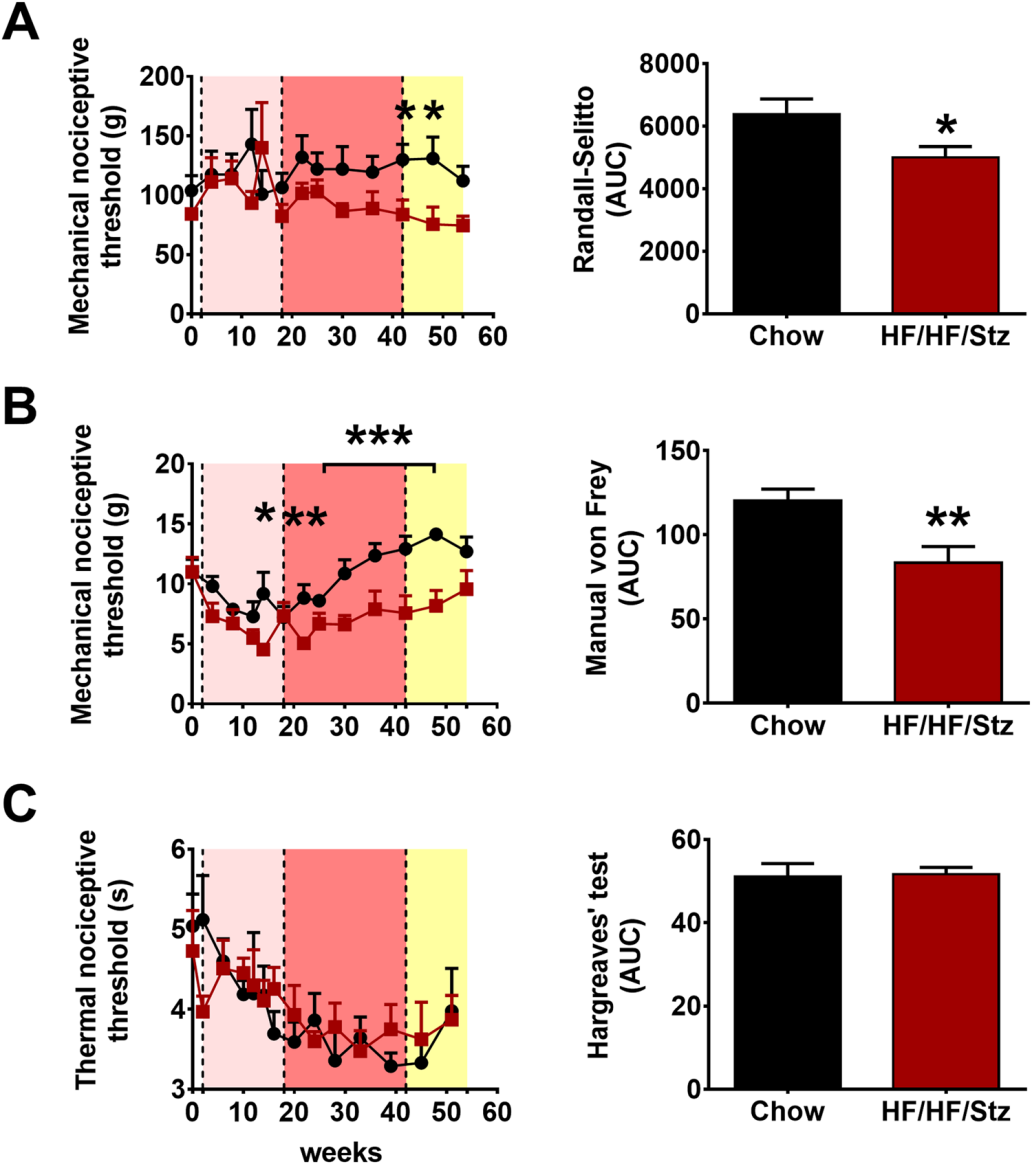
Effect of HF/HF/Stz regimen on pain thresholds. Mechanical hyperalgesia (Randal-Selitto test, (**A**), mechanical allodynia (von Frey hair test, (**B**) and noxious thermal thresholds (plantar test, **C**) are determined over the entire 56-week treatment period. A two-way ANOVA followed by a Holm-Sidak multiple comparisons test is used to compare the time courses. Differences between area under the curve (AUC) are analyzed by two-tailed Mann-Whitney U-test. Error bars represent the SEM. *P < 0.05; **P < 0.01 and ***P < 0.001 compared to rats fed with a chow-diet.

Peripheral neuropathy is a prevalent, disabling complication of diabetes. Here, we examined whether HF/HF/Stz rats exhibited behavioral signs of diabetic peripheral neuropathy, by measuring the changes in mechanical and thermal sensations. Assessment of mechanical hyperalgesia using the Randall and Selitto method revealed that the mechanical nociceptive threshold in HF/HF/Stz rats started to decrease at week 30 and remained lower than in the chow group until week 54 (Fig. 7A, P < 0.05). Accordingly, we observed that HF/HF/Stz rats displayed profound tactile allodynia. Indeed, the paw withdrawal threshold to varying forces of von Frey filaments was significantly lower in diabetic animals compared to normal chow-fed rats, starting at week 18 until the end of the experiment (Fig. 7B, P < 0.01). However, no changes in responses to thermal stimuli were observed using the Hargreaves’ method (Fig. 7C).

**Figure 8:**
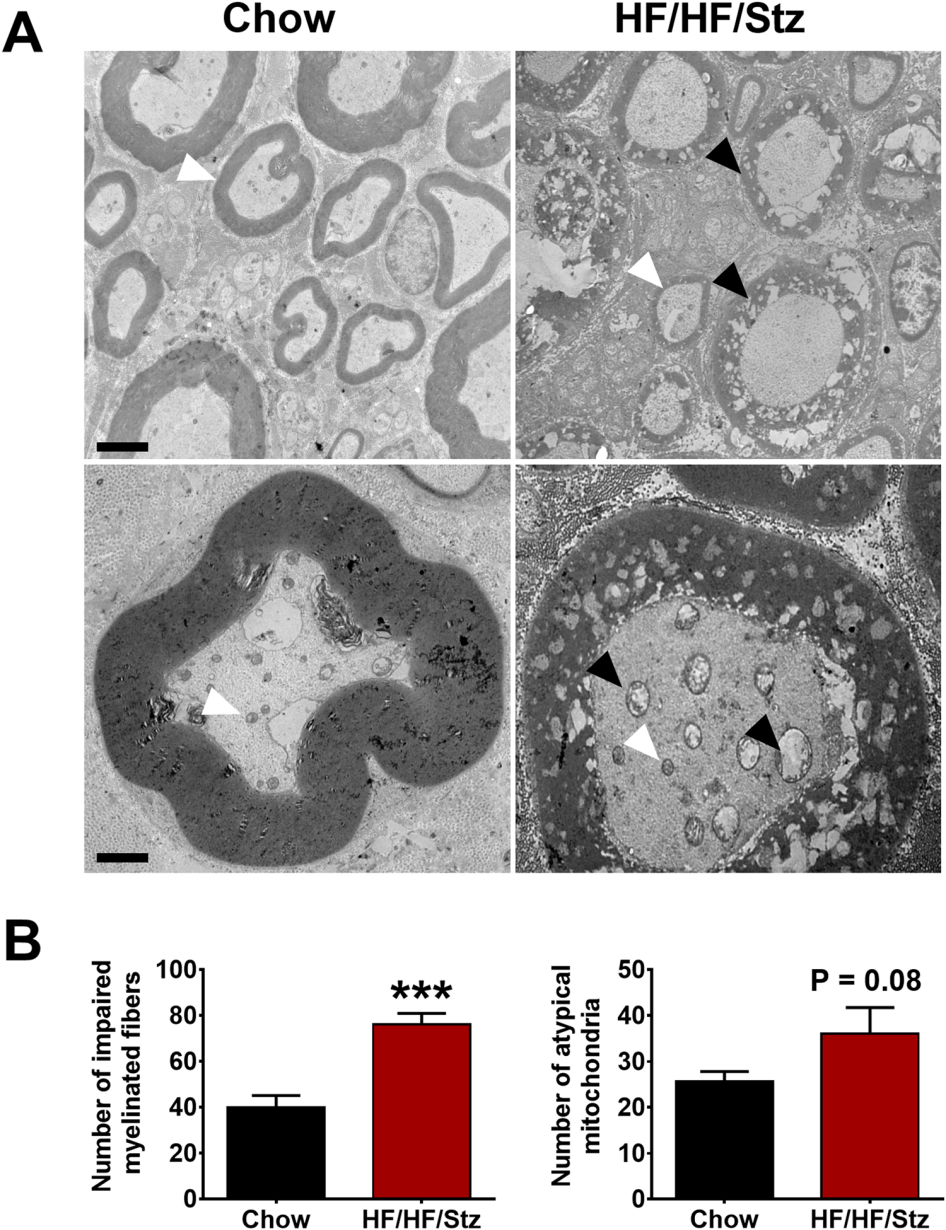
Effect of HF/HF/Stz regimen on the development of diabetic peripheral neuropathy. The peripheral nerve integrity is affected in rats submitted to the HF/HF/Stz regimen, as observed by the demyelination of medium/large fibers in sciatic nerves. White arrows correspond to normal fibers whereas black arrows indicate impaired fibers (**A**; upper panel, scale bar 4 µm). Impaired mitochondria are also detected by electron mic lower panel,roscopy. White and black arrows represent normal and atypical mitochondria, respectively (**A**; scale bar 4 µm). Quantitative analysis further confirms the loss of myelinated fibers and the presence of abnormal mitochondria (**B**). Data are expressed as mean ± SEM; ***P < 0.001, compared with chow fed rats; analyzed using a two-tailed Mann-Whitney U-test.

Since loss of large myelinated fibers may lead to disturbance of light touch sensation, we also examined whether these painful symptoms were correlated with axonal degeneration in nerve fibers. Using electron microscopy, we found a significant increase in impaired myelinated fibers in sciatic nerves of HF/HF/Stz rats, compared to controls (Black arrows in upper panel; Fig. 8A,B, P < 0.001). These abnormalities in nerve fibers were also associated with apparent mitochondrial dysfunction, as observed by the presence of atypical mitochondria (Black arrows in lower panel, P = 0.08, Fig. 8A,B).

### Effects of HF/HF/Stz regimen on the spinal neuronal and glial activation

**Figure 9:**
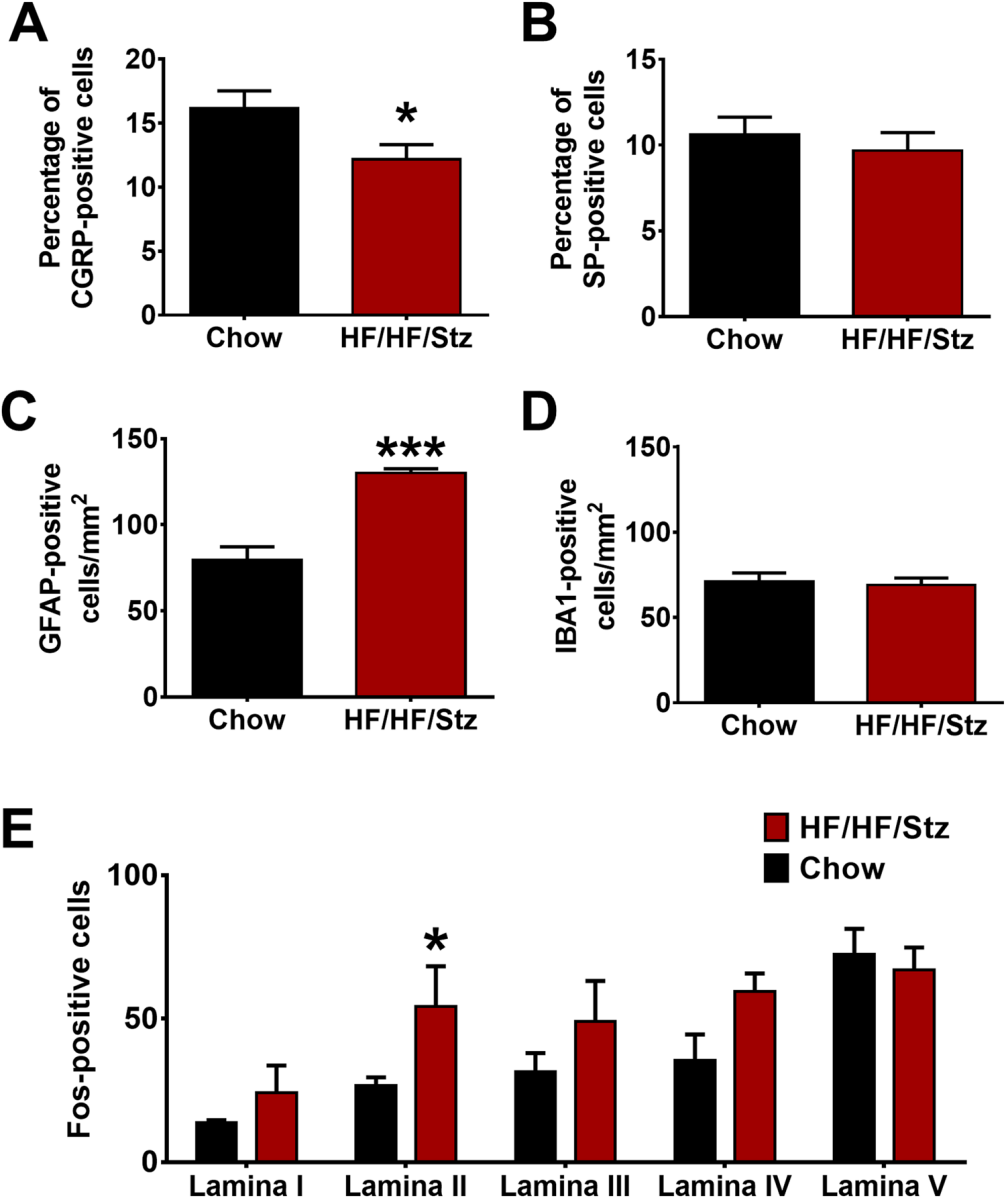
Effect of HF/HF/Stz regimen on neuronal and glial activation in dorsal root ganglia and spinal dorsal horn. Within DRG, CGRP-immunoreactive neurons (**A**), but not SP-labeled cells (**B**) are decreased in the HF/HF/Stz group. Within the spinal cord, the HF/HF/Stz regimen increases the number of GFAP-labeled astrocytes (**C**) but not the number of IBA1-positive microglial cells (**D**). Moreover, the HF/HF/Stz regimen induces a significant increase in Fos expression within lamina II, used here as a marker of nociceptive neuronal activation (**E**). Error bars represent the SEM. *P < 0.05, ***P < 0.001, compared with the chow-diet group. Data (**A**,**B**,**C** and **D**) are analyzed using a two-tailed Mann-Whitney U-test whereas data from (**E**) are analyzed using a Kruskal-Wallis test followed by the Dunn’s multiple comparisons test.

To determine the possible involvement of neuropeptides in the development of abnormal pain in diabetes, we next examined the pattern of distribution of substance P (SP) and CGRP in dorsal root ganglia (DRG) of normal and diabetic rats. We found that the percentage (%) of CGRP-immunoreactive sensory neurons in DRGs of HF/HF/Stz rats was significantly decreased compared to controls (Fig. 9A, P < 0.05). The % of SP-positive cells was, however, similar in both groups (Fig. 9B).

Reorganization of the neuronal-glial network at the cord-level has also been shown to participate in the development and maintenance of various chronic pain conditions. Here, we found that the number of GFAP-positive astrocytes was significantly increased in the spinal dorsal horn of HF/HF/Stz rats, when compared to non-diabetic specimens (Fig. 9C, P < 0.001). In contrast, we did not observe spinal microglia activation, as determined by IBA1 immunostaining (Fig. 9D). These changes in glial reactivity were also accompanied by neuronal hyperactivity. Indeed, higher numbers of Fos-immunoreactive neurons were detected in the spinal dorsal horn of diabetic animals, but in a more pronounced manner within the superficial laminae (Fig. 9E, P < 0.05).

### Effects of HF/HF/Stz regimen on brain function

**Figure 10:**
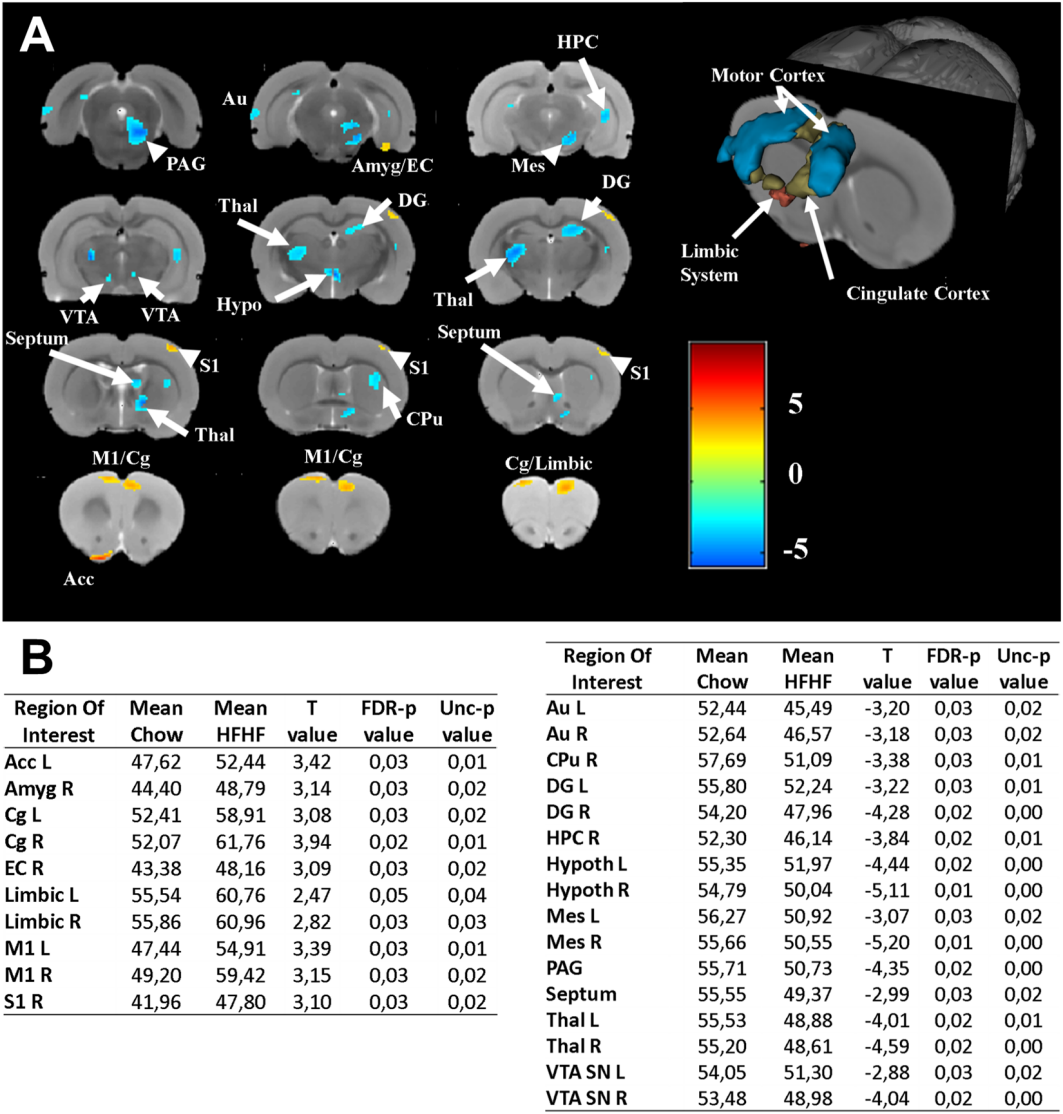
Effect of HF/HF/Stz regimen on brain function. Using µPET imaging, we measured brain consumption of [^18^F]-FDG in HF/HF/Stz and chow-fed rats. Our group analysis allows us to generate statistical parametric t-maps (**A**), where different brain territories display increase (**B**, left panel) or decrease (**B**, right panel) of [^18^F]-FDG uptake. PET data were analyzed using the global linear model implemented in the Statistical Parametric Mapping (SPM) software to generate t-score map (t_(9)_ = 2,47 P < 0.05 False Discovery Rate (FDR) correction). Blobs correspond to P < 0.05 FDR-corrected with an extended threshold of 100 voxels. These regions include the accumbens nucleus (Acc), right amygdala (Amyg), entorhinal cortex (EC), primary somatosensory cortex (S1), cingulate cortex (Cg), primary motor cortex (M1), limbic system (Limbic), periaqueductal grey matter (PAG), caudate putamen (CPu), ventral hippocampus (HPC), auditory cortex (Au), mesencephalic nucleus (Mes), dentate gyrus (DG), hypothalamus (Hypoth), thalamus (Thal), ventral tegmental area/Substantia Nigra (VTA SN). L and R stand for left and right brain.

Finally, we investigated whether the HF/HF/Stz regimen induced changes in regional brain glucose metabolism, as measured by [^18^F]-FDG-PET imaging (Fig. 10A, t_(9)_ = 2,47 P < 0.05 FDR-corrected). Our results demonstrated that several brain structures, including the accumbens nucleus, amygdala, limbic system, entorhinal and primary somatosensory cortices as well as the cingulate and primary motor cortices displayed significant increases in cerebral glucose consumption in HF/HF/Stz rats, compared to chow-fed animals (Fig. 10B, left panel). In contrast, a decrease in glucose consumption was measured in the periaqueductal grey, septum, caudate putamen, and hippocampal formation as well as in the hypothalamus, thalamus, auditory cortex, mesencephalic nucleus and substantia nigra/ventral tegmental area (Fig. 10B, right panel).

## Discussion

Data gathered in clinical trials present compelling evidence to suggest that added sugar and especially added fructose provided from high-fructose corn syrup are posing a serious and growing public health problem, worsening the epidemic of type 2 diabetes^1,8,9^. We thus initiated this study with the objective of developing a unique non-genetic type 2 diabetic rat model adapted to the current diet and lifestyle that would emulate the metabolic features of human T2DM as well as the natural history of the disease progression, from insulin resistance to pancreatic β-cell dysfunction.

The major finding of our study is the characterization of a suitable preclinical model of T2DM, closely mimicking the onset and advanced stage complications of the disease. Indeed, the prolonged HF/HF feeding associated to repeated small-dose Stz every 6 weeks during 20 weeks gives rise to a tri-phasic metabolic adaptation: an early prediabetic phase characterized by an hyperinsulinemic period with modest dysglycemia and without development of marked obesity or dyslipidemia that characterizes a large fraction of patients with T2DM (initial 18 weeks), followed by a late stage of T2DM with frank hyperglycemia, normalization of insulinemia, hepatic fibrosis and marked dyslipidemia (week 18 to 42), and then by spontaneous improvement of blood glucose level (week 42 to 54). HF/HF-fed, STZ-injected rats also developed common symptoms of T2DM such as polyphagia, polydipsia and polyuria. By week 54, HF/HF/Stz diabetic rats exhibited signs of pancreatic β-cell failure, a *sine qua non* condition for the development of T2DM^13^. Indeed, we have documented a marked reduction of the number of pancreatic islets. However, these islets demonstrated marked increase in volume, suggesting compensatory hypertrophic remodeling^31^. Accordingly, recovery of diabetes has been described in a neonatal mouse model treated with low-dose Stz, due to β-cell regeneration^32^. Fructose-enriched diets also caused more pronounced hyperglycemia and hyperlipidemia than high-fat diets^20,23,24^, thus leading to hepatic complications in HF/HF/Stz rats, as determined by measuring the collagen content. The development of this insulin resistance in the liver may result from the lower control of fructose metabolism by insulin or the adipokine leptin. Altogether, this alternative model, involving both insulin resistance and beta cell dysfunction, is useful for studying the pathogenesis of diabetes complications, such as diabetic cardiomyopathy, nephropathy, neuropathy and retinopathy.

Cardiovascular complications are the leading causes of diabetes-related mortality and morbidity. Prominent features of the diabetic myocardium include cardiac hypertrophy, myocardial interstitial fibrosis and apoptosis, left ventricular dysfunction, abnormal electrical conduction and sudden cardiac death^33^. Recent attention has focused on excess fructose consumption as having a unique role in the increase of cardiovascular risk^9^. Here, HF/HF/Stz rats displayed profound reduction in myocardial glucose utilization, as demonstrated by the significant reduction of myocardial [^18^F]-FDG uptake. As published previously^11^, we also found using [^11^C]-acetate PET trends toward reduced stroke volume and myocardial oxidative metabolism, that are compatible with the presence of diabetic cardiomyopathy^34^. Accordingly, recent studies demonstrated that diets-enriched in fructose induced cardiac and vascular complications, including left ventricular contractile and endothelial dysfunction, QRS prolongation, decreased conductance velocity and increased arrhythmogenesis^11,29,35^. Finally, we also observed more cardiac fibrosis in the HF/HF/Stz *versus* control group, which is also compatible with diabetic cardiomyopathy condition^36^.

T2DM is also the leading cause of end-stage of kidney disease, although many but not all patients with T2DM develop renal dysfunction^4^. Our renal [^18^F]-FDG PET imaging, combined to histological analysis demonstrated the presence of pathological features observed in human diabetic nephropathy^37^, including glomerular hyperfiltration and hypertrophy as well as mesangial cell expansion. These pathological changes were also accompanied by a profound increase in the albumin/creatinine ratio in urine samples of HF/HF/Stz rats, indicating the presence of an early stage of diabetic nephropathy^38,39^. Despite the fact that dietary fructose has already been found to lead to tubulointerstitial fibrosis^12^, our renal morphology analysis did not reveal the presence of tubulointerstitial injury, as seen in advanced stages of diabetic nephropathy. Recently, a new rat model named Cyp1a1mRen2 targeting renin-2 gene, was shown to develop signs of advanced renal pathology with progressive albuminuria and decline in glomerular rate filtration^40^. Hence, a very interesting way to induce diabetic nephropathy could be based on a specific deletion of candidate genes within glomeruli using Cas9/CRISPR gene editing technology in rats and applying the HF/HF/Stz regimen to generate a more accurate diabetic nephropathy model.

A common complication associated with diabetes is painful or painless diabetic peripheral neuropathy^41^. Furthermore, symptoms of diabetic polyneuropathy manifest earlier and with a higher prevalence in type 2 diabetes than in type 1 diabetes^42^. However, most preclinical models used to study diabetic neuropathy have serious limitations, including the life span of animals or the type and duration of diabetes^43^. Indeed, diabetic animal models rarely show evidence of obvious neuropathy, such as axonal degeneration of peripheral neurons, segmental demyelination, or fiber loss^44,45^. Here, we found that HF/HF/Stz rats exhibited behavioral signs of diabetic neuropathic pain, including significant reduction in the withdrawal threshold to mechanical pressure and development of tactile allodynia. Importantly, our electron microscopy study also revealed that the HF/HF/Stz regimen affects the integrity of large myelinated fibers and leads to an increase in mitochondrial vulnerability. These results are consistent with previous findings showing damage to peripheral nerves in diabetic subjects, including reduced nerve conduction and loss of large myelinated fibers, which are typically described as late events in diabetic patients^46^. These changes at the peripheral level were also accompanied by a functional reorganization within the spinal dorsal horn circuitry, as noted by the increase in c-Fos-immunoreactive neurons within the superficial laminae, reduced number of CGRP-positive sensory neurons and spinal astrocyte activation. Such neuroplastic changes in the complex neuron-glia networks are in accordance with other reports showing dysfunctional CGRP metabolism, astrocytosis and neuronal hyperactivity within the spinal dorsal horn^47–49^. As observed previously with high-fructose fed rats^50^, the HF/HF/Stz regimen also induced microvascular retinopathy. Morphological lesions were indeed evident, including retinal parenchyma thickening and retinal angiogenesis, as clinically reported among patients with T2DM^5^.

Finally, the concept that diabetes affects the central nervous system is recognized since 1922, and nowadays, the term “diabetic encephalopathy” is frequently used to describe cognitive dysfunctions resulting from diabetes. Indeed, compared to the general healthy population, impaired cognitive function is observed in T2DM patients, particularly when they are exposed to complex cognitive tasks^51,52^. Accordingly, brain imaging studies performed in diabetic patients using magnetic resonance imaging and computed tomography have revealed the presence of general brain atrophy and increased occurrence of white matter hyperintensities that are thought to result from small infarcts^53,54^. In addition, measurements of the cerebral metabolic rate of glucose using PET imaging demonstrate that in pre-diabetic or T2DM patients, insulin resistance and cognitive impairment are directly associated with altered cortical glucose consumption^55^. The impact of high fructose intake in promoting cognitive decline has also recently emerged^56^.

In preclinical model of T2DM, there is, however, a significant lack of studies addressing the effects of diabetes on the brain health. As far to our knowledge, our study is the first to provide a whole brain segmentation using µ-PET imaging to finely explore the metabolic activity in discrete brain areas. We found here that HF/HF/Stz rats display similar glucose metabolic alteration within the limbic system as well as cingulate and frontal cortices, as observed in human diabetic subjects. Our results also demonstrate that the glucose consumption decreases in the hippocampal formation supporting previous findings highlighting the presence of cognitive impairment in diabetic rats, such as spatial working memory deficits. Compared to non-diabetic Wistar rats, non-obese insulin-resistant Goto-Kakizaki (GK) rats display reduced spatial memory, associated to a decrease of presynaptic biomarkers in hippocampus and altered expression of genes involved in neurotransmission within cortical brain regions^57,58^. In other hand, the obese BBZDR/Wor type 2 diabetic rat model exhibits signs of neuronal degeneration, reduced presynaptic densities as well as gliosis in the frontal cortex correlated with a down-regulation of the IGF-1 receptor^59^.

In conclusion, we developed and characterized a new model of T2DM that recapitulates several important metabolic features of the natural history of diabetes and several end-organ long-term complications. The main advantage of our model is that it involves two critical aspects of the pathogenesis of T2D, i.e. β-cell dysfunction and diet-induced insulin resistance, without specific gene invalidation or multiple congenic breeding strategies that results in massive obesity or a more purely beta-cell driven metabolic defect. Moreover, the model is easy to breed at relatively low cost, widely available and usable for behavioral or drug testing.

## References

[1] Pandey A, Chawla S, Guchhait P (2015). Type-2 diabetes: Current understanding and future perspectives. IUBMB Life.

[2] Boudina S, Abel ED (2007). Diabetic cardiomyopathy revisited. Circulation.

[3] Daousi C (2004). Chronic painful peripheral neuropathy in an urban community: a controlled comparison of people with and without diabetes. Diabetic medicine: a journal of the British Diabetic Association.

[4] Retnakaran R (2006). Risk factors for renal dysfunction in type 2 diabetes: U.K. Prospective Diabetes Study 74. Diabetes.

[5] Semeraro F (2015). Diabetic Retinopathy: Vascular and Inflammatory Disease. Journal of diabetes research.

[6] Deepa, M., Anjana, R. M. & Mohan, V. Role of lifestyle factors in the epidemic of diabetes: lessons learnt from India. *Eur J Clin Nutr*, 10.1038/ejcn.2017.19 (2017). 10.1038/ejcn.2017.19 28422123

[7] Johnson RJ (2013). Sugar, uric acid, and the etiology of diabetes and obesity. Diabetes.

[8] DiNicolantonio JJ, O’Keefe JH, Lucan SC (2015). Added fructose: a principal driver of type 2 diabetes mellitus and its consequences. Mayo Clin Proc.

[9] Malik VS, Hu FB (2015). Fructose and Cardiometabolic Health: What the Evidence From Sugar-Sweetened Beverages Tells Us. J Am Coll Cardiol.

[10] Stanhope KL (2009). Consuming fructose-sweetened, not glucose-sweetened, beverages increases visceral adiposity and lipids and decreases insulin sensitivity in overweight/obese humans. The Journal of clinical investigation.

[11] Menard SL (2010). Abnormal *in vivo* myocardial energy substrate uptake in diet-induced type 2 diabetic cardiomyopathy in rats. American journal of physiology. Endocrinology and metabolism.

[12] Nakayama T (2010). Dietary fructose causes tubulointerstitial injury in the normal rat kidney. American journal of physiology. Renal physiology.

[13] Skovso S (2014). Modeling type 2 diabetes in rats using high fat diet and streptozotocin. J Diabetes Investig.

[14] King AJ (2012). The use of animal models in diabetes research. Br J Pharmacol.

[15] Yorek MA (2016). Alternatives to the Streptozotocin-Diabetic Rodent. Int Rev Neurobiol.

[16] Islam MS, Wilson RD (2012). Experimentally induced rodent models of type 2 diabetes. Methods Mol Biol.

[17] El-Bassossy HM, Watson ML (2015). Xanthine oxidase inhibition alleviates the cardiac complications of insulin resistance: effect on low grade inflammation and the angiotensin system. Journal of translational medicine.

[18] Lee JS (2015). Histologic and Metabolic Derangement in High-Fat, High-Fructose, and Combination Diet Animal Models. TheScientificWorldJournal.

[19] Pyo YH, Lee KW (2014). Preventive effect of Monascus-fermented products enriched with ubiquinones on type 2 diabetic rats induced by a high-fructose plus high-fat diet. Journal of medicinal food.

[20] Reed MJ (2000). A new rat model of type 2 diabetes: the fat-fed, streptozotocin-treated rat. Metabolism: clinical and experimental.

[21] Albersen M (2011). Functional, metabolic, and morphologic characteristics of a novel rat model of type 2 diabetes-associated erectile dysfunction. Urology.

[22] Sahin K (2007). Effect of chromium on carbohydrate and lipid metabolism in a rat model of type 2 diabetes mellitus: the fat-fed, streptozotocin-treated rat. Metabolism: clinical and experimental.

[23] Srinivasan K, Viswanad B, Asrat L, Kaul CL, Ramarao P (2005). Combination of high-fat diet-fed and low-dose streptozotocin-treated rat: a model for type 2 diabetes and pharmacological screening. Pharmacological research.

[24] Zhang M, Lv XY, Li J, Xu ZG, Chen L (2008). The characterization of high-fat diet and multiple low-dose streptozotocin induced type 2 diabetes rat model. Experimental diabetes research.

[25] Skovso S (2015). Effects of insulin therapy on weight gain and fat distribution in the HF/HS-STZ rat model of type 2 diabetes. International journal of obesity.

[26] Wilson RD, Islam MS (2012). Fructose-fed streptozotocin-injected rat: an alternative model for type 2 diabetes. Pharmacol Rep.

[27] Leblanc S (2014). Angiotensin II type 2 receptor stimulation improves fatty acid ovarian uptake and hyperandrogenemia in an obese rat model of polycystic ovary syndrome. Endocrinology.

[28] Brassard P (2008). Impaired plasma nonesterified fatty acid tolerance is an early defect in the natural history of type 2 diabetes. The Journal of clinical endocrinology and metabolism.

[29] Lozano I (2016). High-fructose and high-fat diet-induced disorders in rats: impact on diabetes risk, hepatic and vascular complications. Nutr Metab (Lond).

[30] Noll C (2011). Protection and reversal of hepatic fibrosis by red wine polyphenols in hyperhomocysteinemic mice. The Journal of nutritional biochemistry.

[31] Cerf ME (2013). Beta cell dysfunction and insulin resistance. Front Endocrinol (Lausanne).

[32] Kataoka M (2013). Recovery from diabetes in neonatal mice after a low-dose streptozotocin treatment. Biochemical and biophysical research communications.

[33] Huynh K, Bernardo BC, McMullen JR, Ritchie RH (2014). Diabetic cardiomyopathy: mechanisms and new treatment strategies targeting antioxidant signaling pathways. Pharmacol Ther.

[34] Lopaschuk, G. D. Metabolic Modulators in Heart Disease: Past, Present, and Future. *Can J Cardiol* **33**, 838–849, 10.1016/j.cjca.2016.12.013 (2017). 10.1016/j.cjca.2016.12.013 28279520

[35] Axelsen LN (2015). Diet-induced pre-diabetes slows cardiac conductance and promotes arrhythmogenesis. Cardiovascular diabetology.

[36] Bugger H, Abel ED (2014). Molecular mechanisms of diabetic cardiomyopathy. Diabetologia.

[37] Betz B, Conway BR (2014). Recent advances in animal models of diabetic nephropathy. Nephron. Experimental nephrology.

[38] Shang G (2013). 3,5-Diiodo-l-thyronine ameliorates diabetic nephropathy in streptozotocin-induced diabetic rats. Biochim Biophys Acta.

[39] Tan SM (2014). Derivative of bardoxolone methyl, dh404, in an inverse dose-dependent manner lessens diabetes-associated atherosclerosis and improves diabetic kidney disease. Diabetes.

[40] Conway BR (2012). Hyperglycemia and renin-dependent hypertension synergize to model diabetic nephropathy. Journal of the American Society of Nephrology: JASN.

[41] Ziegler D (2009). Painful diabetic neuropathy: advantage of novel drugs over old drugs?. Diabetes Care.

[42] Van Acker K (2009). *Prevalence and impact on* quality of life of peripheral neuropathy with or without neuropathic pain in type 1 and type 2 diabetic patients attending hospital outpatients clinics. Diabetes & metabolism.

[43] Islam MS (2013). Animal models of diabetic neuropathy: progress since 1960s. Journal of diabetes research.

[44] Obrosova IG (2009). Diabetic painful and insensate neuropathy: pathogenesis and potential treatments. Neurotherapeutics.

[45] Gao F, Zheng ZM (2014). Animal models of diabetic neuropathic pain. Exp Clin Endocrinol Diabetes.

[46] Said G (2007). Diabetic neuropathy–a review. Nat Clin Pract Neurol.

[47] Liao YH (2011). Spinal astrocytic activation contributes to mechanical allodynia in a mouse model of type 2 diabetes. Brain research.

[48] Jiang Y, Nyengaard JR, Zhang JS, Jakobsen J (2004). Selective loss of calcitonin gene-related Peptide-expressing primary sensory neurons of the a-cell phenotype in early experimental diabetes. Diabetes.

[49] Morgado C, Terra PP, Tavares I (2010). Neuronal hyperactivity at the spinal cord and periaqueductal grey during painful diabetic neuropathy: effects of gabapentin. Eur J Pain.

[50] Thierry M (2015). Early adaptive response of the retina to a pro-diabetogenic diet: Impairment of cone response and gene expression changes in high-fructose fed rats. Exp Eye Res.

[51] Gradman TJ, Laws A, Thompson LW, Reaven GM (1993). Verbal Learning and/or Memory Improves with Glycemic Control in Older Subjects with Non-Insulin-Dependent Diabetes Mellitus. Journal of the American Geriatrics Society.

[52] Duarte JMN (2015). Metabolic Alterations Associated to Brain Dysfunction in Diabetes. Aging Dis.

[53] Lunetta M (1994). Evidence by magnetic resonance imaging of cerebral alterations of atrophy type in young insulin-dependent diabetic patients. J. Endocrinol. Invest..

[54] Perros P, Deary IJ, Sellar RJ, Best JJ, Frier BM (1997). Brain abnormalities demonstrated by magnetic resonance imaging in adult IDDM patients with and without a history of recurrent severe hypoglycemia. Diabetes Care.

[55] Baker LD (2011). Insulin resistance is associated with alzheimer-like reductions in regional cerebral glucose metabolism for cognitively normal adults with pre-diabetes or early type 2 diabetes. Archives of neurology.

[56] Lakhan SE, Kirchgessner A (2013). The emerging role of dietary fructose in obesity and cognitive decline. Nutr J.

[57] Duarte JMN, Carvalho RA, Cunha RA, Gruetter R (2009). Caffeine consumption attenuates neurochemical modifications in the hippocampus of streptozotocin-induced diabetic rats. J. Neurochem..

[58] Abdul-Rahman O (2012). Altered gene expression profiles in the hippocampus and prefrontal cortex of type 2 diabetic rats. BMC Genomics.

[59] Sima AAF (2010). Encephalopathies: the emerging diabetic complications. Acta Diabetol.

